# Application of ECC as a Repair/Retrofit and Pavement/Bridge Deck Material for Sustainable Structures: A Review

**DOI:** 10.3390/ma15248752

**Published:** 2022-12-08

**Authors:** Hasan Erhan Yücel, Maciej Dutkiewicz, Fatih Yıldızhan

**Affiliations:** 1 Civil Engineering Department, Engineering Faculty, Niğde Ömer Halisdemir University, Niğde 51240, Turkey; 2 Faculty of Civil and Environmental Engineering and Architecture, Bydgoszcz University of Science and Technology, 85-796 Bydgoszcz, Poland; 3 Civil Engineering Department, Engineering Faculty, Gaziantep University, Gaziantep 27310, Turkey

**Keywords:** engineered cementitious composites (ECC), repair, retrofit, pavement, bridge deck

## Abstract

Concrete structures cannot efficiently perform their functions over time due to chemical and physical external effects. Thus, enhancing the relationship between repair and aged structures, and also improving the durability properties of concrete is crucial in terms of sustainability. However, high costs, negative environmental effects, and incompatibility problems occur in repair/retrofit applications. Furthermore, three-quarters of the failures in the repaired/retrofitted structures are caused by a lack of repair durability. The need for repair in pavement/bridge decks is also frequently encountered, and early-age performance problems with repair materials cause pavement/bridge decks to be unavailable for certain periods of time. Engineered Cementitious Composite (ECC) can be effectively used as repair/retrofit and pavement/bridge deck material. It also has a minimal need for repair/retrofit thanks to its high durability properties. This article presents state-of-the-art research regarding the application of ECC as a repair/retrofit and pavement/bridge deck material. Studies in the literature show that the repair/retrofit properties of ECC outperform conventional concrete and steel fiber-reinforced concrete. ECC can be a solution to high early strength and drying shrinkage problems frequently encountered in the use of repair materials. It could also be used for different repair applications such as cast, sprayed, and trenchless rehabilitation. Moreover, ECC might fulfill specific requirements for pavement, pavement overlay, tunnel pavement, airfield pavement, and bridge deck. These superior performances are attributed to ECC’s kink-crack trapping mechanism, uniquely large inelastic strain capacity, strain hardening, high tensile strain capacity, and multiple microcracking and ductile behaviors, especially bonding behavior and self-healing.

## 1. Introduction

Concrete is the most widely used construction material around the world, thanks to its low cost, on-site or precast production, and high compressive strength [1,2,3,4,5]. However, due to its brittleness, low tensile strength, poor toughness property, small fracture energy, and wide cracks, concrete structures are damaged over the years by different modes, such as shrinkage, freeze and thaw cycles, sulfate-other chemical attack and corrosion [1,6,7]. Eventually, these structures become unable to perform their functions. Because it is not feasible to reconstruct damaged structures, the concrete needs to be repaired or retrofitted. Repairing concrete has profound economic, environmental, and compatibility effects, though not as much as reconstruction. For example, in the U.S.A., the cost of maintenance and repair of reinforced concrete highway bridges because of corrosion is up to USD 4 billion annually [8,9]. Materials such as epoxy used in repair cause adverse environmental effects. Also, delamination and swelling can occur due to a lack of compatibility between the repair material and the concrete substrate [8]. Therefore, enhancing the relationship between repair and aged structures and/or improving the durability properties of concrete is crucial in terms of sustainability.

Although repairing the aged structure seems to be the most rational solution [10], many problems occur in its application and many factors need to be considered. The costs of repeated repairs could exceed the initial design and construction costs of the structures [11,12]. For example, in the 1990s, U.S. spending on infrastructure repair and reconstruction was around USD 300 billion [6]. In addition, epoxy, silicone-based polymers, and additional reinforcement applications used for repair/retrofit damage the environment and also cause problems such as bonding and poor durability in the beam-column joint [8,13,14]. Studies show that three out of four failures in repaired elements are caused by a lack of repair durability [15,16]. Repair failure in concrete occurs as a result of physical, chemical, and mechanical events [11,17,18]. One of the main reasons for poor durability from repair applications is non-uniform drying shrinkage [15,16]. Although a low w/b ratio and high-strength concrete are used in repair applications, cracking and delamination easily occur due to the effect of drying shrinkage and high brittleness [19]. Because of cracking and delamination caused by stress between the repair material and concrete substrate, chlorides, oxygen, moisture, alkali or sulfate penetrate easily, thus accelerating deterioration [15,19]. In addition, early-age performance problems in repaired constructions cause them to be unavailable for a certain period of time, especially on pavements [11,20,21]. Long-term durability problems cause a benefit/cost uncertainty [11,20]. In order to increase the performance of the aged structure, repair should be applied with alternative materials that can show higher durability, be more environmentally friendly, and have fewer life costs. In addition, the longer life of the structures produced with higher durability can significantly minimize problems by reducing the need for repair/retrofit. Many researchers have investigated solutions to these problems and Engineered Cementitious Composite (ECC), which can provide both repair/retrofit properties efficiently and also has a minimal need for repair/retrofit thanks to its high durability properties, has been emphasized in these studies [22,23,24].

ECC, which is also known as ultra-high toughness cementitious composites (UHTCC) or strain-hardening cementitious composites (SHCC), is a special type of high-performance fiber-reinforced cementitious composite designed using fracture mechanics and micromechanical principles [25,26,27,28]. Micromechanics allows optimization of the composite for high performance and also minimizes the amount of reinforcing fibers (generally less than 2–3%) [25]. ECC is essentially composed of cement, fly ash, fine sand, polyvinyl-alcohol (PVA) fiber or different fibers (such as polyethylene, polypropylene, polyester, acrylic, nylon, steel, and wollastonite microfibers), water and superplasticizer [27,28,29,30,31,32,33,34,35,36]. Owing to the absence of coarse aggregate and the high cost of fiber, ECC also has a negative environmental impact and high-cost disadvantages [37]. Despite these disadvantages, ECC has extraordinarily mechanical characteristics. ECC shows a strain-hardening behavior after the first cracking [38] and achieves a tensile strain capacity of 3–5%, about 300–500 times higher than conventional concrete [27,30,39,40,41]. Moreover, it has a high ultimate tensile strength (5–10 Mpa), modulus of rupture (8–25 Mpa), fracture toughness (25–30 kJ/m^2^), and compressive strength (up to 80 MPa) [25]. ECC also has good post-cracking resistance and ductility because of the bridging effect of the fibers [42]. After the first cracking, a multiple-cracking pattern is observed. These cracks are saturated distributed multiple microcracks of less than 2 mm, and cracks with a controlled maximum crack width under 200 μm or even 60 μm [43,44]. These superior properties give ECC extensive applicability as repair/retrofit material and durable structure material [42,45].

In addition to its use as a repair/retrofit material, ECC is important and its usage fields are increased because it has superior bonding performance [43], self-healing features [4], and shotcrete use [26], and can be used for trenchless rehabilitation of drinking and sewer infrastructures [46,47] and in pavements [48], which decreases concrete usage and provides architectural diversity [43]. In this study, the use of ECC as repair/retrofit material and its application as pavement and bridge deck were examined in consideration of studies in the literature. In addition, as can be understood from the literature in general, its bond behaviors and self-healing features that directly affect its durability properties are emphasized in the parameters that affect performance the most in ECC applications. Previous studies were systematically examined and their findings were interpreted. Finally, the most important findings are presented here and suggestions for future research are made.

## 2. Use of ECC as a Repair and Retrofit Material

**Figure 1:**
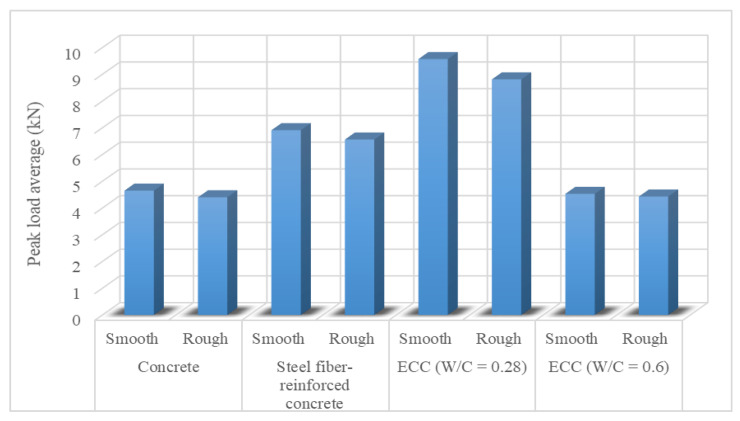
Peak load of the specimens [25].

The fracture behavior, tensile strength, tensile crack, and ductility characteristics of ECC make it appropriate for repair use, thanks to its superior features. Kamada and Li [25] investigated the effect of surface preparation on the fracture behavior of ECC. Three surfaces were prepared as smooth, smooth with lubricant, and rough. ECC with two different w/c ratios, concrete and steel fiber-reinforced concrete, was used as repair materials. Figure 1 shows the peak load of the repair materials. ECC performed the best and this was attributed to the kink-crack trapping mechanism. The kink-crack trapping mechanism includes kinking of the interfacial microcrack into the ECC and arrest in ECC. For ECC, smooth surfaces gave better results than rough surfaces because smooth surfaces had interface crack propagation and this was contrary to the traditional approach to concrete. ECC with a low w/c ratio provided the desired properties better than a high w/c ratio. Wang et al. [6] evaluated the tensile bond strength of ECC repair materials. The influence of roughness, strength of substrate concrete, primer, bonding agent, and also curing age were considered. Four different materials were tested, namely cast-in-situ ECC, prefabricated ECC, cast-in-situ steel fiber-reinforced concrete, and cast-in-situ new concrete. Increasing the surface roughness of the concrete and increasing the substrate strength enhanced the bond strength. This could be attributed to how the rough surface can increase the particular surface area of the old concrete substrate and improve the interlocking between the cast-in-situ ECC and concrete, but there was a certain limit for roughness. Fly ash, silica fume, expansion agent, and styrene-butadiene copolymer (SBR) latex positively affected the primer and bonding agent, and also enhanced bonding strength. The reason could be that fly ash and silica fume can make material components compatible with ECC. The tensile bonding strength is enhanced with curing time. The long-term bonding strength of ECC repair materials was superior to other repair materials. Ahmadi et al. [7] tried to repair tension cracks in concrete panels with high-strength epoxy, glass fiber-reinforced polymer laminate, ECC with slag, and ECC with fly ash. ECC with fly ash and ECC with slag repair materials restored the strength of the damaged panel. In addition, it considerably improved the cracking and leakage. This can be attributed to strain hardening, high tensile strain capacity, and the multiple microcracking behaviors of ECC. This makes ECC a suitable alternative for the repair of reinforced concrete structures, such as water tanks, where superior cracking and leakage are crucial. ECC with fly ash had a lower tensile capacity, small cracks, and less leakage rate than ECC with slag. Li and Li [11] examined the ductility performance of three different repair materials: high-early-strength concrete, high-early-strength steel fiber-reinforced concrete, and high-early-strength ECC (HES-ECC). The high tensile ductility property of HES-ECC enabled durable concrete structure repairs with small delamination lengths and tight surface crack widths. This is because the HES-ECC has a uniquely large inelastic strain capacity.

**Figure 2:**
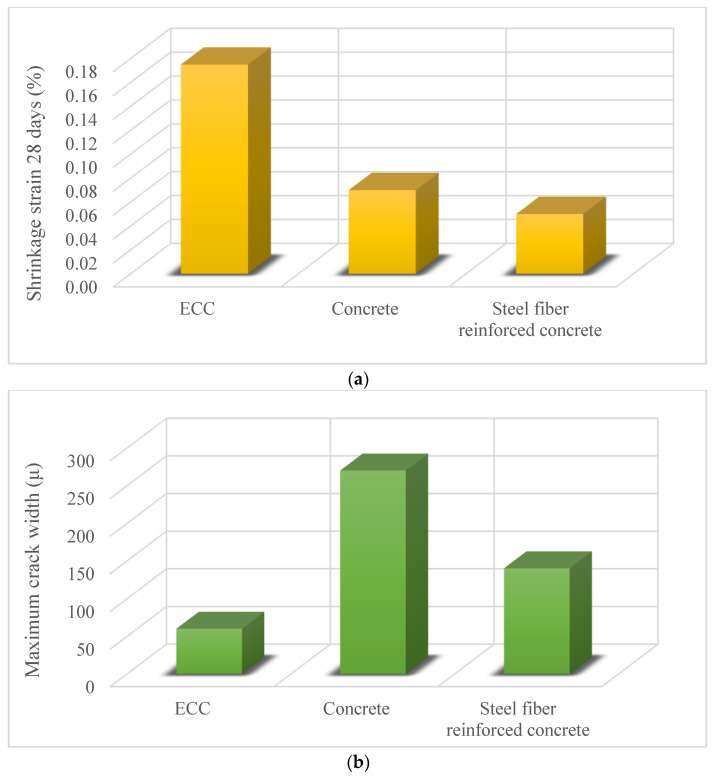
(**a**) Shrinkage strain value (**b**) maximum crack width of repair materials [19].

ECC can be a solution to high-early-strength and drying shrinkage problems encountered in the use of repair materials. Li and Li [21] studied the detailed characterization of high-early-strength ECC (HES-ECC) for concrete repair. HES-ECC and HES-concrete repair materials were compared. The compressive strength of HES-ECC was 23.59 ± 1.40 MPa at 4 h and 55.59 ± 2.17 MPa at 28 days. The flexural strength was HES-ECC 9.81 ± 0.24 MPa at 4 h and 15.08 ± 0.34 MPa at 28 days. The flexural strength of ECC twice exceeds the flexural strength of concrete with similar compressive strength. The strain-hardening behavior and tensile ductility of HES-ECC is the reason for its superior flexural strength. The tensile strength of HES-ECC was 3.46 ± 0.08 MPa at 4 h and 5.68 ± 0.14 MPa at 28 days. Its early high-strength property enables ECC to be used for fast repair. Also, HES-ECC showed superior tensile strain-hardening behavior and self-controlled crack behavior under restrained shrinkage conditions. Matrix toughness and matrix/fiber interface properties positively affect the mechanical properties of ECC. Li and Li [19] tested the shrinkage, cracking, and interface delamination performance of concrete, steel fiber-reinforced concrete, and ECC as a repair material. Figure 2a shows the shrinkage strain of concrete, steel fiber-reinforced concrete, and ECC. ECC had the highest shrinkage strain owing to the absence of coarse aggregate. Steel fiber-reinforced concrete had the lowest shrinkage strain due to throughout influence of steel fibers. Figure 2b shows the maximum crack width of concrete, steel fiber-reinforced concrete, and ECC. The maximum crack width of ECC was 60 μm, which was considerably lower than the other two repair materials. Even though ECC had the highest drying shrinkage strain value, it showed the most desirable repair material properties because of the very small interface delamination. Steel fiber-reinforced concrete was subjected to the most severe delamination. Because the cracking potential is high and these cracks are bridged by steel fibers, therefore they could not open freely. These cracks could then not resist shrinkage deformation, and finally, delamination occurred. The findings of this study show the importance of the drying shrinkage factor for repairing material.

**Figure 3:**
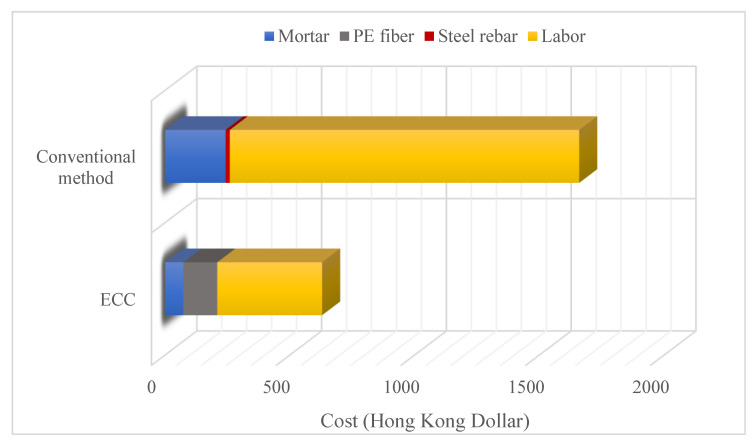
Costs of ECC and conventional method for repairing [14].

ECC can be used in steel corrosion and beam-column joints where repair-retrofitting needs are high. Chen et al. [14] tried to compensate for the loss of load-carrying capacity due to corrosion deterioration, one of the main problems of concrete structures reinforced with ECC. ECC was recommended as repair material because splice additional reinforcement, which is a common technique, is time-consuming and costly. ECC was designed with PE fiber, and five mixes were formed by increasing the sand ratio (tested to some extent as it causes poor fiber dispersion) and reducing the w/b ratio. Tests were performed by repairing the reduced area rebars with ECC. High tensile strength and ultimate tensile stress could be obtained with ECC, which was produced with a very low water/binder ratio and the addition of silica fume. A higher sand ratio increased the tensile strength. ECC fully recovered the load-carrying capacity of the corroded rebar. The cost of ECC and the conventional method for repairing Y20 rebar are given in Figure 3. Repairing with ECC is less costly than the conventional method and equals approximately 38% of the conventional method. Lim et al. [13] studied reinforced concrete beam-column joint retrofitted by carbon fiber reinforced polymer grid covered with ECC and with high-strength mortar. According to the results of the experiments, ECC had higher total energy dissipation and ultimate strength compared to high-strength mortar. The ductile behavior of the ECC provided these advantages. ECC could retain the applied displacement longer, by at least a 0.5 ductility level, before the specimens collapsed. Also, spalling of the high-strength mortar was more than it was for ECC.

ECC is capable of different repair applications such as cast, sprayed, and trenchless rehabilitation. Kim et al. [26] investigated sprayed ECC as a repair material and compared it with prepackaged mortar. Ultimate strain capacities of about 1.6% were obtained from the ECC samples at 28 days and this value was up to 100 times the ductility of prepackaged mortar. Crack widths of the ECC were controlled with an average of 30 μm. According to flexural test results, sprayed ECC had high MOR and large deflection capacity with outstanding energy absorption compared to the prepackaged mortar. Also, ECC showed consistent flexural behavior for the opposite loading direction. Zhu et al. [1] developed a sprayable ECC by using calcined clay-limestone cement, calcium sulfoaluminate, and polypropylene fiber to decrease carbon emission and costs, and increase durability. Developed sprayable ECC had comparable strength, higher tensile strain capacity, and reduced crack width compared to casting ECC. The advantages of developed sprayable ECC were low carbon, low shrinkage, low cost and ultra-high ductility. Thus, developed ECC is encouraging for wider repair applications. One of the ECC usage fields is the repair/retrofit of concrete infrastructures [46,47]. Concrete is one of the most used materials in drinking and wastewater pipelines. Zhu et al. [46] used ECC, spray-in-place, grouting, slip lining, modified slip lining, cure-in-place pipe, close-fit, and fiber-reinforced polymer lining for concrete pipeline trenchless rehabilitation. ECC provided retrofit for concrete pipelines thanks to its ultra-high ductility, tailorable mechanical performance, and finely dispersed microcrack properties. The improvement of load capacity and leak-proof ability thanks to the microcrack-self-healing properties of ECC and it being a resilient material, provided advantages for ECC as a sustainable material. Zhu et al. [47] used centrifugally sprayed ECC to retrofit cracked concrete pipes. According to the test results, its superior mechanical properties, leak-proof performance, low cost, fast construction, and self-healing ability make centrifugally sprayed ECC promising for the rehabilitation of concrete pipelines such as tunnels and culverts, in addition to water infrastructure. The findings of these studies show that sprayed ECC application is convenient for repair/retrofit use [1,26,47]. Crucially, ECC can also be used for trenchless rehabilitation of infrastructure, which is a more effective approach than open-cut [46,47].

**Figure 4:**
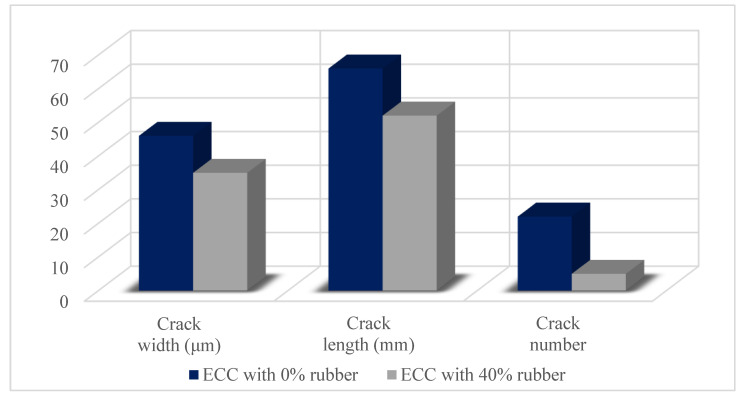
Crack width, crack length, and crack number of ECCs at 30 days [15].

ECC also enables the use of different materials such as recycled, bacteria, superelastic shape memory alloy, and sulfoaluminate cement. Huang et al. [15] tested recycled rubber for ECC repair material. The crack width, crack length, and crack number for specimens containing 0% and 40% recycled rubber are shown in Figure 4. Utilization of tire rubber enhanced the cracking resistance, and reduced the number of cracks and crack initiation time. Also, tire rubber enhanced the tensile ductility and durability of repair ECC. However, the tensile and compressive strength of ECC with tire rubber decreased considerably. This was attributed to an increment in the porosity of ECC. Although this condition limits the use of ECC astructural repair material, it is sufficient for non-structural repair material. It is very valuable to use recycled materials in ECC repair materials to solve the recycling difficulty of waste materials. Beltran et al. [8] examined bio-based ECC repair systems. The mechanical properties of bio-based ECC and its bonding behavior were tested and compared with ECC without bio-based. Bio-based ECC performed adequately in terms of its compressive and bonding strength properties. Bio-based ECC showed less delamination compared to ECC without bio-based. Additionally, bio-based ECC showed a slightly better recovery of both flexural strength and deflection capacity compared to ECC without bio-based. Li et al. [49] studied ECC with superelastic shape memory alloy. This showed remarkable energy dissipation capacity, minimal residual deformation, and full self-recovery of damage. Furthermore, the tensile strain capacity of ECC with superelastic shape memory alloy, tailored up to 5.5%, showed a distributed microcracking feature with microcrack width self-controlled under 40 μm. Li et al. [10] tested ECC based on sulfoaluminate cement and fiber-reinforced polymer. Uniaxial tensile tests and four-point bending tests were performed. According to the test results, ECC based on sulfoaluminate cement and fiber-reinforced polymer layer had a considerable effect on controlling the crack, enhanced superior mechanical and structural durability properties. In another study, Pan et al. [50] stated that fiber-reinforced polymer grid-reinforced ECC repairing/strengthening performance was better than ECC. Zhou et al. [51] expressed that ECC with polyethylene has great potential for structural strengthening and repair due to its great tensile strength, flexural strength, fatigue performance, ductility, strain-hardening performance, post-peak mechanical properties, and impact resistance. Studies in the literature show that ECC is a more sustainable material than other materials because it can solve the durability problems of structures effectively.

## 3. Use of ECC as Pavement and Bridge Deck

**Figure 5:**
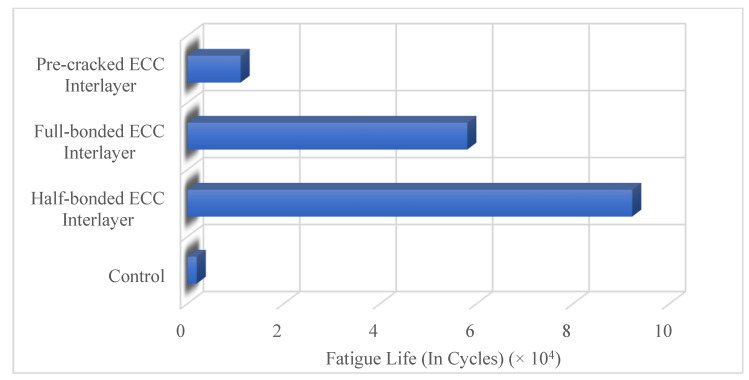
Fatigue life of control and ECC mixtures [57].

While durability and bond behavior are significant features for repair/retrofit material [22,50], its load-bearing capacity, low maintenance requirement, ductile characteristics, and penetration resistance are significant features for pavement/bridge deck material [48,52]. The outstanding properties of ECC materials allow them to be used in transportation infrastructure. Moreover, the life cycle cost of ECC is lower than conventional concrete and hot mix asphalt because of reduced energy consumption and greenhouse gas emissions [53,54]. This is because ECC requires fewer major and minor repairs and has a longer service life. Arce et al. [55] tried to develop low PVA fiber content ECC by incorporating non-oil coated PVA fiber, fine river sand, and fly ash for jointless pavements. According to the test results, PVA-ECC with low fiber content was developed using non-oil-coated PVA fiber, fine river sand, and fly ash. The low fiber content decreased the flexural strength, tensile strength, and tensile ductility of ECC, but compressive strength increased. This increment was attributed to a decreased probability of fiber lumps formation and an increase in material density because of the removal of fibers. Also, incorporating fly ash increased the ductility of the mixture, but strengths were negatively affected. Although the fatigue performance of the developed mixture was worse than ECC, it was better than conventional concrete. The findings of this study showed that brittle failures in rigid pavements can be mitigated, and thickness reduction and cost-effectiveness can be achieved with the developed low-fiber content ECC. Bawono et al. [53] investigated the low skid resistance and surface drainage of ECC pavement applications due to the absence of coarse aggregates in ECC. Corundum was used instead of sand to enhance the surface microtexture. Incorporating corundum reduced the tensile strain capacity. This was attributed to corundum with high hardness. Corundum increased the matrix toughness, decreased the strain hardening and multiple cracking potential. However, tensile strain capacity was adequate for pavement applications. The utilization of corundum improved the micro-texture and skid resistance of ECC, and its durability properties, but the enhancement of macrotexture and surface water drainage were marginal. Bawono et al. [56] evaluated bright and slip-proof ECC with visible light-activated photo-catalysis properties for pavements in tunnels. A carbon-modified TiO_2_ was used to activate a visible light-activated photo-catalysis property. In addition, white fine aggregates were incorporated to enhance skid resistance. White ECCs containing 20% silica sand and 5% TiO_2_ had compressive strength > 50 MPa, tensile strength > 4.5 Mpa, and tensile strain capacity > 4%. In addition, it had a high skid resistance of approximately 80 British pendulum number (BPN) and high surface reflectivity with a lightness value of 90. According to the findings of this study, ECC can provide specific properties and functions adequately in tunnel pavement applications. Wu et al. [52] developed innovative cement-based composite pavement consisting of three layers including asphalt concrete, high-strength concrete, and ECC. According to the study results, a thicker ECC layer would increase the bending resistance of the pavement and decrease the penetration depth. The ductile performance of ECC could be the reason for low penetration. In addition, tension cracks at the bottom of the ECC layer were remarkably decreased. Wu et al. [52] used ECC as the bottom layer, while another study used ECC as an interlayer [57]. Das et al. [57] tested ECC as a new ductile interlayer to increase resistance to reflective cracking. A thin ECC interlayer was constructed between Portland cement concrete and hot mix asphalt overlay. According to the test results, the ECC interlayer successfully mitigated the reflective cracking and changed the failure mode of the pavement. The fatigue lives of the control and ECC mixtures are given in Figure 5. Specimens with ECC interlayers showed considerably (30 to 47 times) higher fatigue life compared to control specimens (without ECC interlayers). This could be attributed to the post-cracking ductile behavior of ECC. The findings of this study showed that the ECC interlayer is capable of mitigating reflective cracking.

**Figure 6:**
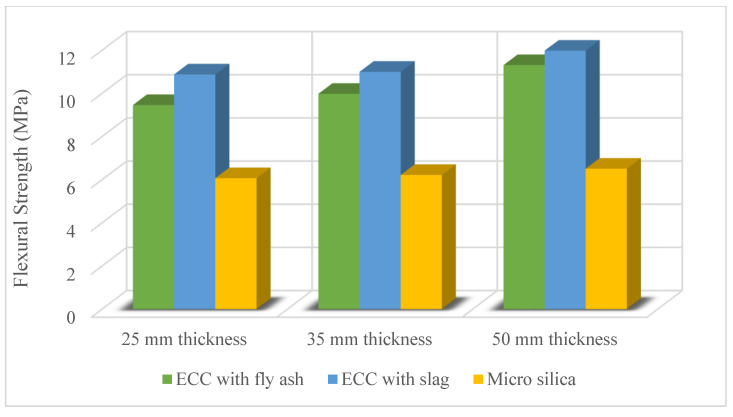
Flexural strength of ECC mixtures and micro-silica concrete at 28 days [48].

ECC can be used as a pavement overlay because it has a high load-carrying capacity and low maintenance costs. Yucel et al. [48] evaluated the ECC overlay system for the rehabilitation of rigid concrete pavement and compared it with micro-silica concrete. Figure 6 shows the flexural strength of the ECC mixtures and micro-silica concrete at 28 days. ECC with 25 mm thickness outperformed micro-silica concrete with 50 mm thickness. Therefore, ECC overlay can be produced much more thinly than micro silica concrete and shows both more economic and superior performance. The ECC enhanced load-carrying capacity and deformability. Also, ECC exhibited better crack width control compared to micro-silica concrete. ECC was found to be an effective overlay material by mitigating reflective cracking and delamination. Yücel [58] examined the behavior of high-volume Class F fly ash and slag incorporated ECC as overlay materials under cyclic freezing and thawing effects and compared them with silica fume concrete. ECC mixtures safely performed their function of 300 freeze-thaw cycles without any failure, while only one out of six silica fume concrete survived 300 freeze-thaw cycles. Also, ECC with slag performed better than ECC with Class F fly ash. Qian et al. [59] studied ECC overlay performance from a life cycle point of view and compared it with unbonded concrete overlay and hot mix asphalt. Results from life cycle analysis showed that ECC overlay has better performance than unbonded concrete overlay and hot mix asphalt due to reduced thickness, long service life, and/or less frequent repair requirement. Pranav et al. [60] performed an economic input-output life cycle analysis of ECC pavement overlay by incorporating corundum. Compared to full-depth concrete and ECC pavement overlay, corundum-blended ECC overlay pavement was a more sustainable material for almost all the parameters (economic impacts, energy consumption, environmental inventory, etc.) of the life cost analysis. Studies show that corundum-blended ECC could be an alternative pavement and/or pavement overlay composite material [53,60]. Moreover, ECC pavement overlay has not only superior mechanical and durability properties but also many advantages for life cycle assessment [59,60]. Hungria et al. [61] evaluated a novel jointless ECC ultrathin whitetopping overlay. Their results showed that ECC ultrathin whitetopping overlay provides considerable cost savings compared to regular jointed concrete ultrathin whitetopping overlay. Potential jointless and decreased thickness properties accounted for the majority of the construction cost savings. Also, studies in the literature demonstrate that ECC is a more sustainable material than other materials since it requires fewer repairs and has a longer service life.

ECC can also be used in airfield pavement thanks to its superior mechanical properties. Ma and Zhang [24] proposed an ECC overlay on concrete airfield pavement for mitigating reflective cracks. The results of the study showed that ECC overlay could enhance the load-bearing capacity of concrete airfield pavement considerably and delay reflective cracks. Furthermore, ECC overlay increased the fatigue life of airfield pavement compared to Portland cement concrete. Studies show that both the use of ECC interlayer as pavement and the use of ECC overlay as airfield pavement can mitigate reflective cracks [24,57]. Wu et al. [62] examined the abrasion resistance and acoustic wave attenuation of ECC for airfield pavement. It was found that the abrasion resistance of ECC could be comparable with concrete of the same compressive strength. Moreover, it was stated that the acoustic wave attenuation of ECC was higher than ordinary concrete having the same compressive strength [62]. Pan et al. [63] studied ECC with glass fiber-reinforced polymer mesh reinforcements for runway pavement and investigated impact fatigue behavior. Glass fiber-reinforced polymer mesh-reinforced ECC pavement specimens could sustain 30,000 impacts without failure while concrete pavement specimens failed after only several impacts because of macro cracks.

One of the use fields of ECC is bridge deck, as ECC provides the specific requirements that it must have. Ma et al. [64] studied medium early strength (MES)-ECC as steel bridge deck pavement material. According to the test results, the compressive strength of MES-ECC at three days reached 26 MPa and the flexural strength reached 8 MPa. Moreover, MES-ECC had high ductility. These results show that the use of ECC can shorten the traffic opening time of the steel bridge as well as the construction period. Chu et al. [65] tested the fatigue behavior of glass fiber-reinforced polymer-reinforced ECC link slabs. According to the experiment results, glass fiber-reinforced polymer-reinforced ECC link slabs have a greater post-fatigue residual rotational capacity and a lower rate of stiffness degradation. Also, glass fiber-reinforced polymer-reinforced ECC link slabs enhanced the post-fatigue retention of the energy-absorbing capacity. Wu et al. [66] used ECC as steel deck pavement, incorporating magnesia oxide (MgO) and superabsorbent polymer (SAP) to improve the self-healing and bonding properties. Utilization of MgO and SAP decreased the micro defects of the interface and enhanced the durability, self-healing, and bonding properties. Furthermore, the initial cracking strength of ECC increased slightly. Studies have shown, therefore, that ECC can be used as pavement, pavement overlay, tunnel pavement, airfield pavement, and bridge deck, and also it can satisfy the specific requirements for these uses.

## 4. Effective Properties Providing ECC Application for Repair/Retrofit and Pavement/Bridge Deck

ECC shows superior performance in many properties, such as mechanical, ductility, and durability. In this section, the bond behavior and self-healing properties of ECC, which are the most effective ECC properties and provide superiority in the use of repair/retrofit and pavement/bridge deck, were briefly explained in light of studies in the literature.

### 4.1. Bond Behavior of ECC

**Figure 7:**
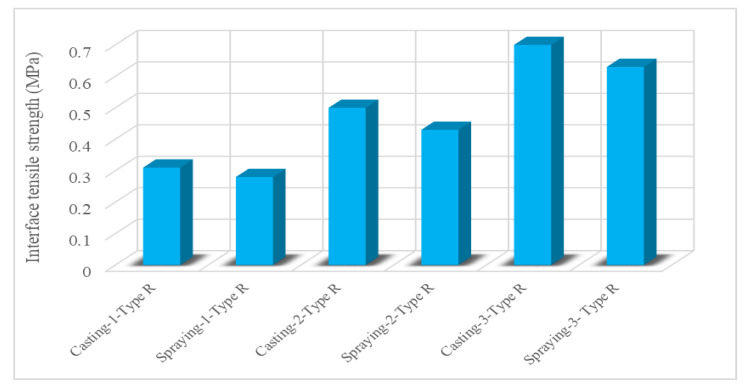
Effect of construction methods on interface tensile strength of ECC mixtures [28].

**Figure 8:**
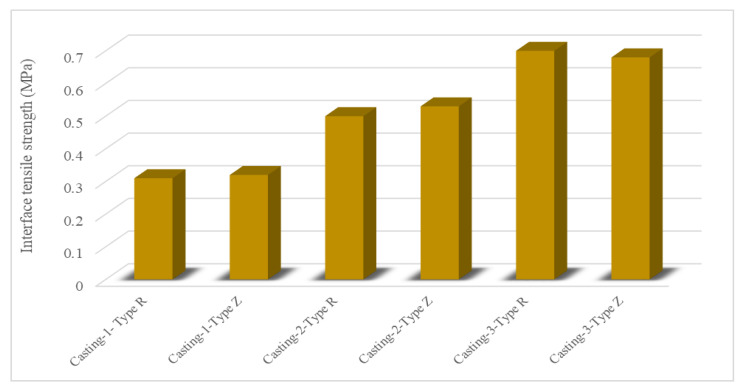
Effect of PVA fiber type on tensile strength of ECC mixtures [28].

Bond is defined as the interaction between the reinforcing steel (or substrate material) and the surrounding concrete (or repair material). This interaction allows the transfer of tensile stress from the steel (or substrate material) to the concrete (or repair material) [67]. The bonding performance of ECC is discussed in the literature from different aspects and positive observations have been made. The superior bonding performance of ECC was attributed to its extraordinary mechanical properties. Zhou et al. [42] examined the bond behavior between ECC and steel bar, considering the effects of ECC strength, steel bar diameter, and fiber-reinforced polymer (FRP) confinement. Increments in the strength of ECC affected the bond strength of the specimen positively but affected the maximum slip negatively. Moreover, increments in the diameter of the steel bar caused a decrease in the maximum bond strength. The strength of ECC had a substantial effect on the effectiveness of FRP confinement. For specimens with lower ECC strength, circumferential confinement was not used effectively and no substantial effect on the bond-slip curves could be observed. For specimens with higher ECC strength, an increment in circumferential confinement had a notable effect on bonding behavior, causing maximum and residual bond strengths, and also maximum slip. Lastly, there was a limit to the improvement of the bond behavior through circumferential confinement. Sahmaran et al. [68] investigated the bond strength between ECC overlay and a conventional concrete substrate. Microsilica concrete (MSC) was used as a control mixture. ECC overlay increased bond strength remarkably compared to microsilica concrete. According to the results of the slant shear test, the bond-strength properties of the layered ECC substrate concrete were greater than the strength of substrate concrete which has a compressive strength of approximately 30 Mpa while layered MSC substrate concrete failure consistently took place at the interface. Tian et al. [28] studied the tensile performance of ECC-to-concrete interface with both an experimental study and a mechanical model and considered the effects of the construction method, the compressive strength of ECC, PVA fiber type, and interface roughness. The interface roughness and compressive strength of ECC had a significant effect on the failure mode while the construction method and PVA fiber type slightly affected the failure mode. The effect of different construction methods on the tensile strength of ECCs is given in Figure 7. Casting and spraying refer to the construction method, and Type R refers to the PVA fiber type in Figure 7. The construction method, compressive strength of ECC, and interface roughness had a significant effect on the nominal bond strength of the interface. Spraying ECC affected the nominal interface strength negatively. Increments in interface roughness and the compressive strength of ECC affected the interface bond strength positively. The effect of PVA fiber type on the tensile strength of ECCs is given in Figure 8; type R and type Z refer to the PVA fiber type. PVA fiber type had little effect on interface bond strength. Chang et al. [69] evaluated the bond behavior of magnesium phosphate cement-based ECC by considering material proportion (water-solid mass ratio, sand-to-binder mass ratio, molar ratio of MgO to KH_2_PO_4_, fly ash content, borax content, and PVA fiber volume fraction). According to single and double shear bond strengths, increments of sand to binder mass ratio, the molar ratio of MgO to KH_2_PO_4_, and the volume fraction of the PVA fibers affected the strength positively and the solid mass ratio negatively. An increment of the fly ash content affected the single shear bond strength positively up to 30% and then negatively; the double shear bond strength was negatively affected. Increments in borax dosage affected the single and double shear bond strengths positively first and then negatively.

Carbon fiber reinforced polymer (CFRP) is used for repair thanks to its high strength and durability properties. Zhang et al. [70] tested the (CFRP)-ECC-concrete composite interface with a single shear test. According to the test results, it was stated that the ECC layer could delay debonding. For specimens with an ECC layer, the average bearing capacity, ultimate slip, and ultimate strain were higher than the specimen without an ECC layer. Increments in the thickness of the CFRP plate and concrete/ECC strength had a positive effect on bearing capacity. Zhang et al. [71] investigated CFRP plate-ECC-concrete composite interface specimens under freeze-thaw cycles with a single shear test. According to the findings from the study, the CFRP plate-ECC-concrete composite interface can effectively delay the peeling of the CFRP plate. Also, CFRP plate-ECC-concrete composite interface transfers the shear stress more effectively. Freeze-thaw cycles affected the bonding performance of the composite interface. Tensile strength, compressive strength, and bearing capacity decreased after the freeze-thaw cycle. In addition to the freeze thaw cycle, the effect of high temperature was also investigated [72]. In this study [72], bonding properties between the existing concrete and ECC exposed to high temperature were examined. In the first specimen (Type A), the existing concrete was first heated and then repaired with ECC and strengthening concrete. In the second mixture (Type B), the existing concrete was first reinforced with repair material and then heated. Slant shear and the splitting tensile test were performed. For Type A, below 200 °C, slant shear and the splitting tensile increased, and the bonding properties were enhanced as the temperature increased. Above 200 °C, slant shear and the splitting tensile and bonding properties decreased as the temperature increased. The bonding performance of the existing concrete/ECC was better than the existing concrete/strengthening concrete. For Type B, a temperature increase to 600 °C had a positive effect on the bonding performance of the existing concrete/ECC. Also, a burst occurred in the existing concrete/strengthening concrete at 600 °C while no burst occurred in the existing concrete/ECC even at 800 °C.

### 4.2. Self-Healing Property of ECC

**Figure 9:**
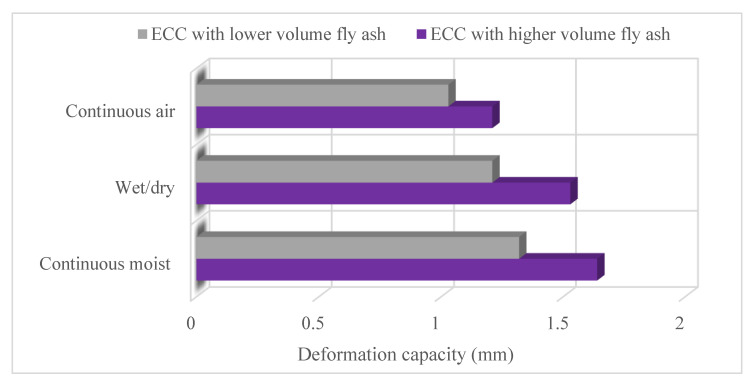
Deformation capacity of ECC mixtures [77].

Self-healing is defined as the ability of a material to detect and repair internal damage without any outside interference [4,73]. The self-healing mechanism is attributed to the hydration of previously unhydrated cementitious material, swelling of C–S–H, calcite formation, the closing of cracks by impurities within the water, and the spalling of loose concrete particles [74,75]. As a result of these events, the closure of cracks is explained as self-healing. ECC has tight crack properties that are many and near to each other; thus it has superior self-healing properties because tight crack is the key factor for self-healing [75]. Zhang and Zhang [76] examined the influence of 60 °C hot water and Ca(OH)_2_ solution on the self-healing behavior of ECC. According to the test results, Ca(OH)_2_ solution and 60 °C hot water considerably increased the compressive strength, fly ash reaction degree, and hydration product. Therefore these environmental conditions could improve the self-healing property of ECC. Also, 60 °C hot water is more effective than Ca(OH)_2._ Özbay et al. [77] tested the self-healing properties of ECC containing high-volume fly ash. A splitting tensile strength test was performed to generate microcracks in ECC specimens; these specimens were then exposed to further continuous wet, continuous air, and wet/dry conditions. Figure 9 shows the deformation capacity of the ECC mixtures. Utilization of high-volume fly ash enhanced the self-healing properties because of a tighter crack width and a higher amount of unhydrated cementitious material. Continuous moist and wet/dry conditions affected the self-healing properties of ECC positively, enhanced its mechanical properties, and remarkably decreased its rapid chloride permeability. Sahmaran et al. [75] studied the effects of different mineral admixtures on the self-healing properties of ECC under various environmental exposure conditions. Low-calcium fly ash, high-calcium fly ash, and slag were used as mineral admixtures. According to the test results, the type of mineral admixtures remarkably affected the self-healing properties of ECC. ECC specimens containing fly ash had more non-hydrated cementitious materials and higher self-healing capacity. According to the findings of these studies, the reaction products associated with mineral admixtures have a major effect on the self-healing properties of ECC and ECC and enable its use for high amounts of industrial waste (fly ash, etc.) [75,77]. Özbay et al. [78] investigated the effects of sustained flexural loading on the self-healing properties of ECC. Flexural loading was performed to generate microcracks and then the ECC was stored under continuous water or air exposures with or without sustained mechanical loading. Specimens in continuous water without sustained mechanical loading showed flexural deflection capacity recovery because of the healing products in the microcracks, whereas this condition was not seen in air-exposed specimens. C-S-H gel from the continued hydration of unhydrated cement and fly ash was observed filling microcracks in continuous water. Moreover, when sustained mechanical loading was performed on the pre-cracked specimens during the exposure period, recovery of mechanical properties was decreased thanks to the self-healing properties. Qiu et al. [79] evaluated self-healing on the flexural fatigue performance of ECC. According to their results, self-healing majorly extended the fatigue life of the ECC. Water/dry conditioning healed matrix cracks and also recovered fiber/matrix interfacial bonds, which led to increased fiber bridging strength. Another study showed that the superior self-healing feature of ECC is not only evident in the controlled laboratory environment but also in the natural environment [74]. These studies indicate that the self-healing properties of ECC are opportune for further advancement. The self-healing properties can be attributed mainly to the tight-crack width and high cementitious material content [78].

## 5. Conclusions

This study presented the use of ECC as a repair/retrofit and pavement/bridge deck material. Furthermore, the bonding behavior and self-healing properties, which provide superior performance for a repair/retrofit and pavement/bridge deck, were also presented. After considering studies in the literature, the following results can be drawn:

- For repair/retrofit use, the fracture behavior performance of ECC is better than concrete and steel fiber-reinforced concrete thanks to the kink-crack trapping mechanism. The tensile bond strength and ductility performance of ECC are satisfying because of the uniquely large inelastic strain capacity. This makes ECC a suitable alternative for the repair of reinforced concrete structures, such as water tanks, where superior cracking and leakage are crucial.
- ECC can be a solution to the high-early-strength and drying shrinkage problems frequently encountered in the use of repair materials. ECC outperforms concrete and steel fiber-reinforced concrete in terms of cracking and interface delamination.
- ECC could be used in steel corrosion and beam-column joints for repair/retrofit. The repair cost using ECC is cheaper than the cost of concrete, equalling approximately 38% of the cost of concrete.
- ECC is convenient for different repair applications such as cast, sprayed, and trenchless rehabilitation. Crucially, ECC can also be used for trenchless rehabilitation of infrastructure, which is a more effective approach than open-cut.
- ECC enables the use of different materials, such as recycled, bacteria, superelastic shape memory alloy, and sulfoaluminate cement. The utilization of tire rubber enhances ductility and durability, but the tensile and compressive strength of ECC with tire rubber decreases. The use of waste materials is also crucial for sustainability.
- For pavement/bridge deck use, the life cycle cost of ECC is lower than conventional concrete and hot mix asphalt because of reduced energy consumption and greenhouse gas emissions.
- The overlay performance of ECC is quite adequate. ECC with 25 mm thickness is better than micro-silica concrete with 50 mm thickness. Therefore, the economic life cycle analysis of ECC pavement is more sustainable than concrete overlay.
- ECC can be used as pavement, pavement overlay, tunnel pavement, airfield pavement, and bridge deck, and it also satisfies the specific requirements for these uses. ECC mitigates reflective cracks, and enhances load-bearing capacity and fatigue life. In addition, abrasion resistance and acoustic wave attenuation performance are sufficient level.
- ECC has superior bonding behavior properties. Bond behavior of ECC-steel bar, ECC overlay-conventional concrete substrate, and the tensile performance of ECC-concrete interface is satisfactory. Furthermore, bonding performance of ECCunder different conditions such as freeze-thawing and high temperatures is adequate.
- ECC has also outstanding self-healing properties. High amounts of industrial waste (fly ash, etc.) positively affect the self-healing properties of ECC. Flexural loading on the self-healing properties of ECC and self-healing on the flexural fatigue performance of ECC are satisfactory.
- ECC is a more sustainable material than other materials because it can solve the durability problems of structures effectively, requires fewer repairs, and has a longer service life.

In future studies, different waste materials can be tested to increase sustainability and examine the effects of different materials on ECC. The effect of inert materials (such as wollastonite, etc.) on repair/retrofit and the pavement/bridge deck performance of ECC can be examined and even tested in combination with waste material. Cost analyses can be performed using studies that reduce the PVA or PE fiber content, which constitutes the majority of ECC costs.

## Data Availability

Not applicable.
